# Invasive and Non-Invasive Approaches of Electrical Stimulation to Improve Physical Functioning after Spinal Cord Injury

**DOI:** 10.3390/jcm10225356

**Published:** 2021-11-17

**Authors:** David R. Dolbow, Ashraf S. Gorgey, Tommy W. Sutor, Vanesa Bochkezanian, Kristin Musselman

**Affiliations:** 1 Physical Therapy Department, William Carey University, Hattiesburg, MS 39401, USA; 2 College of Osteopathic Medicine, William Carey University, Hattiesburg, MS 39401, USA; 3 Spinal Cord Injury and Disorders Center, Hunter Holmes McGuire VA Medical Center, Richmond, VA 23229, USA; Thomas.Sutor@va.gov; 4 Department of Physical Medicine and Rehabilitation, Virginia Commonwealth University, Richmond, VA 23298, USA; 5 Physiotherapy Department, School of Health, Medical and Applied Sciences, College of Health Sciences, CQ University Rockhampton, Rockhampton, QLD 4702, Australia; v.bochkezanian@cqu.edu.au; 6 Department of Physical Therapy and Rehabilitation Sciences Institute, University of Toronto, Toronto, ON M5G 1V7, Canada; Kristin.Musselman@uhn.ca; 7 KITE, Toronto Rehabilitation Institute, University Health Network, Toronto, ON M4G 3V9, Canada

**Keywords:** rehabilitation, exercise, neurological, spinal cord injuries, paraplegia, tetraplegia

## Abstract

This review of literature provides the latest evidence involving invasive and non-invasive uses of electrical stimulation therapies that assist in restoring functional abilities and the enhancement of quality of life in those with spinal cord injuries. The review includes neuromuscular electrical stimulation and functional electrical stimulation activities that promote improved body composition changes and increased muscular strength, which have been shown to improve abilities in activities of daily living. Recommendations for optimizing electrical stimulation parameters are also reported. Electrical stimulation is also used to enhance the skills of reaching, grasping, standing, and walking, among other activities of daily living. Additionally, we report on the use of invasive and non-invasive neuromodulation techniques targeting improved mobility, including standing, postural control, and assisted walking. We attempt to summarize the effects of epidural stimulation on cardiovascular performance and provide a mechanistic explanation to the current research findings. Future trends such as the combination of epidural stimulation and exoskeletal-assisted walking are also discussed.

## 1. Introduction

Disability following spinal cord injury (SCI) is primarily determined by the level and completeness of the injury. The higher the level of injury, the less sensory and motor activity available for physical tasks, including those related to activities involved with daily living. Likewise, complete injuries limit physical activity abilities more than incomplete injuries. However, other factors can compromise the level of physical functioning, including dramatic changes in body composition and muscle tone. One therapeutic approach with the potential to address the myriad of sensory, motor, and autonomic impairment characteristics of SCI is electrical stimulation. Both non-invasive and invasive applications of electric currents to the body have been developed and studied over the past four decades, resulting in considerable evidence and guidance to support the clinical use of electrical stimulation. In this narrative review, current electrical stimulation applications for individuals living with SCI are summarized to provide a broad perspective of this evolving therapeutic approach.

## 2. Obesity Decreases Functional Mobility

Traumatic SCI induces a critical illness response period where the basal metabolic rate (BMR) and resting metabolic rate (RMR) increase by ~55% [1,2]. During this time, protein is the primary substrate used for energy, with skeletal muscle comprising about 67% of the protein catabolized [1,2]. Muscle loss can be as high as 5% per day after the injury, depending on the severity of the injury [1,2]. These factors confirm decades of research that have demonstrated extreme muscle atrophy within a few weeks of injury [3,4]. This extreme muscle atrophy is accompanied by the infiltration of ectopic adipose tissue known as intramuscular fat (IMF). This contributes to changes in muscle quality and the mechanical properties of the paralyzed muscles [5,6,7]. Recently, a lower muscle stiffness of the vastus lateralis muscle has been reported in persons with SCI compared to health controls, due to the infiltration of IMF [7]. The extensive loss in the contractile myofilaments forces the paralyzed muscle to downsize its metabolic demands by an extensive reduction in mitochondrial density and, subsequently, leading to a decreased basal metabolic rate [8], which has been shown to be a critical consequence to increasing the prevalence of obesity after SCI [9,10].

Once the critical response period has ended, the extensive loss of metabolically active muscle contributes to an RMR of ~25% less than in non-SCI peers. The combination of the low RMR with the low level of energy expended due to decreased mobility can decrease the total daily expenditure to 50% below that of able-bodied individuals [11,12,13]. The altered energy balance equation with typically more caloric intake than expenditure is a primary reason for the reported 97% obesity rate and a mean body fat percentage (BF%) of over 42% in the SCI population [14,15].

Spungen and colleagues studied eight sets of identical twins with one twin in each pair having SCI and determined that the twin with the injury had an average 13.1% greater BF% [16]. Edwards et al. found that those with SCI have a 58% greater trunk visceral adipose tissue than age, gender, and waist circumference-matched able-bodied individuals, along with increased total body fat and visceral fat [17]. Gorgey and Dudley discovered that individuals six weeks post SCI have 126% more intramuscular fat than controls without SCI. Furthermore, at a three-month follow-up, it was found that those with SCI had gained an additional 26% of intramuscular fat [4].

Increased adiposity is likely to continue long-term due to the diminished lean mass coupled with physical inactivity after SCI. Van den Berg-Emons et al. used an accelerometer to demonstrate that the physical activity level of community-dwelling individuals with SCI is only 40% of that of able-bodied individuals [18]. The increases in visceral and intramuscular adiposity have been linked to altered cardio-metabolic profiles in persons with SCI [10,19,20].

Much of the rehabilitation process after SCI is focused on improving functional activities such as bed mobility, dressing, transferring in and out of bed, wheelchair propulsion, and other routine activities of daily life. This is typically accomplished by strengthening muscles above the SCI [21]. However, obesity places a greater burden on the limited available voluntary musculature of the arms, making it more difficult to achieve and maintain functional abilities. This, in turn, diminishes physical independence and community integration [22]. In a study reviewing the records of over 1000 individuals with SCI from six different rehabilitation facilities, it was determined that, for people with paraplegia, the American Spinal Injury Association (ASIA) impairment scores of A, B, and C, being overweight or obese was associated with diminished motor functional independent measures (FIM) [23]. Motor categories in the FIM include self-care activities (dressing, bathing, grooming, toileting, and eating), transfer tasks, and locomotion tasks, including wheelchair propulsion [24]. Stenson et al. also found that obese individuals with paraplegia had significantly lower FIM scores in self-care and mobility than non-obese individuals with paraplegia [25]. Similar findings in earlier case reports by obese individuals with SCI support the premise that obesity is a barrier to meeting and maintaining functional goals [26,27].

## 3. Electrically Evoked Training to Improve Body Composition

Physical activity is commonly prescribed to help manage obesity and the associated health consequences such as cardiovascular and metabolic diseases. Because of the limited available voluntary muscle in the arms, often electrical stimulation activities for the legs are also considered [28]. Neuromuscular electrical stimulation (NMES) and functional electrical stimulation (FES) activities conducted through surface electrodes have been shown to increase muscle mass, decrease fat mass, and decrease BF%, which can help ameliorate the obesity barrier to increased functional abilities and to improve cardiometabolic health.

Gorgey et al. completed a randomized control trial comparing the effects of NMES resistance training and testosterone therapy to the use of testosterone therapy alone in 22 individuals with chronic SCI over 16 weeks [5]. The results showed that the NMES plus testosterone group significantly increased in the total body lean mass, whole muscle mass, and knee extensor cross-sectional area, while the testosterone-only group showed no changes. A 14–17% increase in BMR also highlighted the potential importance of increasing lean mass for producing a healthier body composition. The basis of the work originated from earlier works that showed that effectively loading the paralyzed muscles results in robust muscle hypertrophy close to 40% [29,30,31]. Attempts to decipher the molecular mechanism behind this robust muscle hypertrophy [32,33] have demonstrated that muscle hypertrophy is accompanied by increased citrate synthase as a marker of mitochondrial density, succinate dehydrogenase as a mitochondrial enzyme of complex II activity, and an increase in the number of myonuclei [33,34]. Muscle hypertrophy is also accompanied by an improvement in muscle quality as cauterized by decreased IMF, increasing specific tension and slowness in the rise time [5,6]. Rise time reflects the mechanistic properties of different fiber types, and the slow rise time may serve as a physiological indication of a fiber-type transformation from the fatigable fast glycolytic fibers to the fatigue-resistant slow oxidative fibers [6].

Demchak et al. used an FES leg cycling exercise (LCE) 4–6 weeks after SCI on 10 acutely injured individuals three times per week at a cycling cadence of 35 revolutions per minute (rev/min^−1^) [35]. After 13 weeks, the cross-sectional area of the vastus lateralis muscle increased 63% more than the non-stimulated control exercise leg. This indicates that FES applied early after injury can attenuate muscle loss. Dolbow et al. investigated the effects of high-intensity interval training FES cycling three times per week for eight weeks combined with a 30 min weekly nutritional counseling session on 10 adults with chronic SCI [36]. The group that performed FES cycling three times per week and received nutritional counseling once per week demonstrated a 5.7% increase in leg lean mass and a 2.4% decrease in BF%, while the group receiving nutritional counseling only, slightly decreased in leg lean mass and increased in BF%. However, while there is significant evidence that electrical stimulation activities can be successful in helping to restore a more favorable body composition, questions remain concerning specific parameters that may optimize these and other results.

## 4. Optimizing Electrical Stimulation Training Parameters

Fornusek et al. investigated the differing effects between high and low-cadence FES cycling. One leg performed FES cycling at a 10 rev/min^−1^ cadence, while the other leg performed a cadence of 50 rev/min^−1^ [37]. Both legs significantly increased in thigh girth, demonstrating muscle hypertrophy. However, after six weeks of training three times per week, the low-cadence leg demonstrated a significantly greater girth circumference and electrically evoked isometric torque. A similar observation was noted following 6 months of FES cycling on muscle volume, trabecular bone parameters, and biomarkers of bone turnover in adults with SCI. Participants in the low-cadence group cycled at a maximal average torque of 2.9 ± 2.8 Nm and the high-cadence group cycled at a maximal average torque of 0.8 ± 0.2 Nm. Low-cadence cycling showed greater decreases in bone-specific alkaline phosphatase. N-telopeptide decreased 34% following low-cadence cycling, suggesting a decrease in bone resorption. Both groups increased muscle volume (low-cadence cycling by 19%, high-cadence cycling by 10%). Low-cadence cycling resulted in a 7% non-significant increase in the apparent trabecular number and a 6% decrease in the apparent trabecular separation in the distal femur without noticeable changes in the high-cadence group [38].

Gregory and Bickel described the motor fiber recruitment pattern with electrical stimulation as non-selective and asynchronous, resulting in the early onset fatigue of the stimulated muscle groups [32]. Deley et al. studied the comparison of variable electrical frequencies and continuous frequency training in 10 individuals with SCI and found significantly less training fatigue with variable frequency training than continuous training [39]. This may indicate that interval training options are more effective for individuals with SCI. Gorgey et al. investigated the effects of three different pulse widths (200 µs, 350 µs, and 500 µs) during FES cycling over three weeks [40]. There was no difference in the peak oxygen uptake (VO_2_ peak) or energy expenditure (EE). However, exercising at 350 µs resulted in a greater difference between FES cycling and resting than at 200 µs. Gorgey et al. summarized that FES cycling at 350 µs may be better for EE. However, Gorgey also found that 500 µs elicited an autonomic dysreflexia response, while the lower levels did not. The authors also studied the effects on peak torque and muscle fatigue at different frequencies that ranged from 20 to 100 Hz. They noted that peak torque dropped by more than 50% after an acute bout of FES cycling and remained down by more than 20% following 72 h. of cycling, suggesting low frequency fatigue [41]. Based on these findings, the authors suggested to space the frequency of the training to twice weekly especially in chronic persons with less training experience in FES cycling [41].

Bochkezanian et al. used a different approach to the use of NMES investigating the effects of high-intensity strength training performed under isometric conditions using a lower frequency (30 Hz) and a higher pulse width (1000 µs) for 12 weeks on five adults with chronic SCI [42]. The participants were seated with the hip and knee joint angles at 85 and 90 degrees, respectively. Electrical stimulation was delivered to the legs separately every 20 s, starting at an electrical stimulation intensity of 30 mA and then increasing by 10 mA incrementally until a plateau in observed maximal peak twitch or 99 mA was reached. The results showed that after 12 weeks, the muscle strength and quadriceps cross-sectional area were significantly increased and subjective measures of spasticity were also ameliorated [42].

While no conclusions concerning optimal specific protocols have been determined, the evidence does support that, for NMES, higher intensity resistance training produces strong muscle contractions that result in significant gains in muscle strength and muscle mass in individuals with SCI [42]. Concerning FES-LCE, evidence has demonstrated that a lower cycling cadence and greater muscle stimulus can increase lean mass. The addition of nutritional counseling has also demonstrated benefits for battling obesity [30,35,36,37].

## 5. Management of Spasticity

The use of surface neuromuscular electrical stimulation (NMES) resistance training (RT) has previously been identified as an effective rehabilitation strategy to manage spasticity in people with SCI [43], and some studies suggested that it may reduce spasticity levels [42,44]. A recent systematic review investigating the effects of electrical stimulation parameters to ameliorate symptoms of spasticity in people with SCI concluded that the use of NMES can reduce spasticity by 45–60%. This was evidenced with electromyography activity and an increase in the range of motion. The stimulation parameters utilized for the spasticity management were variable with a 20–30 Hz frequency, current amplitude of more than 100 mA, and pulse durations of 300–350 μs [45].

## 6. Clinical Applications of FES/NMES

In terms of the clinical applications of FES/NMES, the use of FES cycling is increasingly being used routinely in the clinical rehabilitation setting for people with SCI. Some of the optimal electrical stimulation parameters require the use of high cadence (40–50 rev/min^−1^) and monitoring of the rate of perceived exertion (RPE) and Heart Rate (HR) peak at a moderate level for the purposes of aerobic fitness and light level for cardiometabolic benefits [46]. Conversely, to improve muscle strength, the cadence should be low (10–20 rev/min^−1^) to allow the principles of progressive strength training, such as progressively increasing the load by adjusting the current intensity, and increasing the evoked force and training volumes [47,48]. Additionally, a recent systematic review of health and fitness-related outcomes in people with SCI concluded after analyzing 92 studies that FES-cycling has a moderate–high certainty for improvements in muscle health, referring to muscle mass and fiber-type composition, whereas they found a low certainty (using the GRADE rating) for the power output and aerobic fitness outcomes category, although all 35 studies investigating the power output and aerobic fitness reported significant improvements [49].

FES as a strength training modality is less common in clinical practice, but the benefits have been extensively proven in the literature [5,28,30,42]. Some of the evidence concerning the optimal dosage for muscle strength purposes includes a total time of 35 min, two to three times per week for at least 6 weeks and up to 3 years in duration and a wide pulse width (500–1000 μs) [9,40,42,50,51] and low to moderate frequencies (20–30 Hz) to prevent contraction-induced muscle injury (Black et al., 2008). Additionally, 48 to 72 h between sessions was shown to be adequate to allow for full muscle fatigue recovery [40].

## 7. Retraining Standing Postural Control with FES

Beyond NMES resistance training and FES-LCE, FES is used to enhance the skills of reaching, grasping, standing, and walking, among other activities of daily living [52,53]. Effective postural control is required for the successful completion of most activities of daily living; however, individuals living with both motor complete and incomplete SCI experience significant deficits in postural control. Two important components of standing postural control are challenged after SCI. First, functional stability limits, which involve the ability to move the center of mass within a base of support [54,55], are impaired. Individuals with motor incomplete SCI have reduced control of their center of mass when standing, resulting in smaller functional boundaries [56,57,58]. Second, reactive postural control, which involves the ability to return the center of mass to within the base of support using corrective motor strategies after a loss of balance [54,55], is also impaired in individuals with SCI during both standing [59] and walking activities [60]. The consequences of this impaired postural control are living with a high risk of falling and experiencing frequent falls, both of which can have significant negative impacts on physical and psychosocial well-being [61,62].

To date, postural control has rarely been a therapeutic target of FES. Yet, there is emerging evidence that FES can be incorporated into balance exercises for individuals living with motor incomplete SCI. More specifically, FES has been used to facilitate the retraining of functional stability limits. A closed-loop FES system was recently combined with visual feedback balance training in a standing position [57]. Visual feedback balance training is a commonly used therapeutic approach that provides greater feedback to an individual concerning the location of their center of pressure in space to safely learn to move within their base of support. The training involves the individual standing on pressure sensors while viewing a computer screen that shows the location of their center of pressure. FES can facilitate the completion of visual feedback balance training by targeting the dorsiflexor and plantar flexor muscles, which are critical for controlling the movement of the center of mass in a standing position [63]. The triggering of surface electrical stimulation to the dorsiflexors and/or plantar flexors is automated; the system continually monitors the individual’s center of pressure location and stimulates the correct muscle group involved in moving the center of mass to within the base of support or to another area as instructed by the therapist. Houston et al. (2020) used FES to retrain functional stability limits in five individuals with incomplete SCI three times per week for four weeks, and found clinically relevant increases on the Berg Balance Scale and mini-Balance Evaluation Systems Test (mini-BESTest). Participants also demonstrated an increased center of pressure excursion in all directions when leaning in a standing position [57].

Gauthier et al. (2020) recently demonstrated the feasibility of incorporating FES into perturbation-based balance training, which involves repetitively exposing individuals with SCI to external perturbations to help retrain reactive postural control [64,65,66]. Surface electrodes were placed on the common fibular nerve bilaterally to elicit the flexor withdrawal response. During this study, involving two individuals with motor incomplete SCI, a stimulation was triggered by pressure-sensitive footswitches. As the investigator applied an external perturbation (i.e., manual push or pull) to cause the individual’s center of mass to move outside of their base of support, the participant’s pressure came off the footswitch, initiating the electrical stimulation to assist with limb flexion, initiating a reactive step. This step widens the base of support allowing the center of mass to once again be within the base of support. The training involved three one-hour sessions per week for six weeks, with each session including 30–40 perturbations. The demonstrated benefits were an increased walking speed, increased mini-BESTest Total Score, and increased mini-BESTest Reactive Score [64].

An important consideration in the applications of electrical stimulation is the effectiveness of the stimulation parameters. We have recently provided in-depth details of the stimulation parameters that are likely to increase muscle mass, enhance the body composition profile, and reduce spasticity in persons with SCI [45,65]. The current work primarily focused on applications of electrical stimulations towards enhancing motor control; however, other indications similar to pain management, bladder, rectal, and sexual dysfunctions have been previously highlighted [67].

## 8. Improved Function through Less Invasive and Invasive Electrical Stimulation

**Figure 1:**
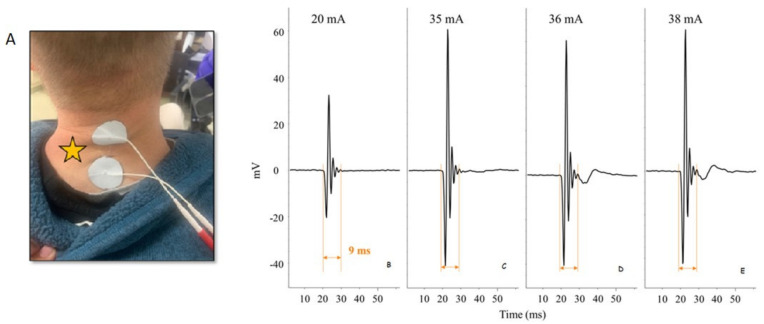
Details of determining the amplitude of TSS current delivered during intervention sessions. Spinal-evoked potentials elicited by the Theratouch stimulator of the biceps brachii obtained from a non-injured volunteer in our lab are shown. Overall, panels (**B**–**E**) show baseline EMG activity and stimulation artifact occurring at a consistent time length. Electrodes were placed at the C3–C4 and C6–C7 intervertebral spaces, and on the lateral clavicles as shown in (**A**); 2 ms biphasic stimuli were delivered in a configuration that the intervertebral electrodes functional as the cathode upon change in polarity of the biphasic stimulus at 30 Hz. Panels (**A**,**B**) show no EMG response following stimulation at different intensities. Panel (**D**) shows an evoked response at a current (36 mA) that was only one mA different from that in panel (**C**), which evoked no response (35 mA). Panel (**E**) confirms the consistent EMG response, at a consistent latency, at higher amplitudes. Thus, in this example, motor threshold would be considered 36 mA, and this individual would complete intervention procedures with TSS delivered at a submotor threshold of 27 mA (75% of motor threshold).

In addition to the non-invasive applications of electrical stimulation, less invasive applications have emerged in rehabilitation and become considerably attractive for restoring motor functions and other vital functions in persons with SCI. The first form is trans-spinal stimulation (TSS) or transcutaneous stimulation, and relies on dorsal applications of electrical current to the interspinous process. Cathodes can be placed externally on the back, with the anodes placed bilaterally on the iliac crests. The electrical current can either be a direct or biphasic current that targets the large dorsal afferent fibers, mainly the proprioceptive fibers (Ia and II) that originate from the muscle spindles and loop into the cord to reflect the status of the contracting or exercising muscles. Although the activation of other afferent fibers similar to the mechanoreceptors III and IV have not been excluded, there is still no suggesting evidence to confirm their activation during TSS. It was suggested that using a medium frequency carrier current would likely facilitate penetration with less activation of the sensory nociceptors. A biphasic current of 30 Hz, pulse duration of 300–1000 µs, and amplitude of the current of 0–200 mA has been recommended [68]. A longer pulse duration close to 1000 µs is the key to successfully activating the neural circuity at the lumbosacral or cervical segments. TSS is likely to neuromodulate the neural network circuitries at the sub-threshold or motor threshold levels. This is likely to increase the level of excitability of the afferent pathways, inter-neuronal proprioceptive networks, and motor pathways. Non-invasive TSS enhances the neuroplasticity of the corticospinal pathways and leads to an improved upper extremity function. Figure 1 highlights the procedure of setting up motor and sub-motor thresholds following establishing the spinal motor-evoked potential of the biceps muscle via application TSS. In this case, the sub-motor threshold was set arbitrary at 75% of the evoked motor threshold. A recent work investigated a similar research question and compared among the 80%, 90%, and 110% spinal motor threshold of the abductor policies brevis muscle [69]. The authors showed that 90% intensity elicited a greater improvement in the F/M ratio, cortical motor-evoked potential, box and blocks test, and maximum voluntary contraction evaluations in the healthy able-bodied population. The authors used a carrier frequency current of 10 kHz that is likely to increase the depth of penetration and reduce the discomfort during TSS [69]. This may explain the choice of using a 75% sub-motor threshold during applications of TSS.

A growing body of evidence supports that TSS of the spinal cord promotes functional recovery in humans with SCI [53,68,70,71,72]. Motor and sensory functional improvements have been observed when TS is applied alone, or in combination with other therapies. Importantly, TSS applied at the cervical level can increase the upper extremity function in people with tetraplegia. After a single session of TSS, Benavides et al. found that TSS had an excitatory effect at the spinal level as measured by cervico-medullary-evoked potentials and an inhibitory effect at the cortical level as measured by motor-evoked potentials [70]. These changes were associated with an improved upper extremity function in people with tetraplegia. An assessment of spinal cord-evoked potentials demonstrated that these results were due to an increased level of spinal network excitability with tonic TSS and training. Gad et al. proposed the use of cervical TSS as a non-invasive approach to modulate the cervical network segments, resulting in an improvement in EMG and torque and ensuring functional recovery following stimulation twice weekly for four weeks [73]. After eight training sessions with TSS, combined with training over four weeks for 1–2 h per session, maximum voluntary handgrip forces increased by 325% in the presence of stimulation and 225% when grip strength was tested without simultaneous stimulation in chronic tetraplegia 1–21 years post-injury. Subjects demonstrated an improved upper extremity function starting from the first training session as demonstrated by their abilities to generate a greater force. Neurophysiologic data suggest that TS can modulate cervical spinal networks into a state that enables greater access to the supraspinal control of cervical motor and sensory networks. Thus, both spinal interneurons and motor neurons are placed closer to the motor threshold and are more likely to respond to a descending voluntary drive [73].

**Figure 2:**
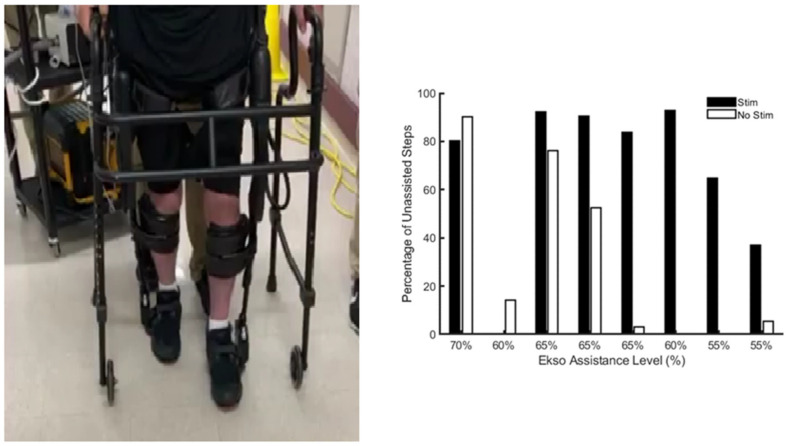
The use of exoskeleton assisted walking with TSS to facilitate stepping at lower level of assistance in a person C6 motor complete SCI. Percentage of unassisted steps during 10 m walking test was determined by the number of steps that the person could imitate on their own without triggering the audible sound of the exoskeleton compared to the total number of steps. Triggering the audible sounds indicated that the robotic exoskeleton initiated the steps. It is typical to have around 16–18 total steps per leg with a swing complete time set at 0.5 s. Only with the TSS, the person with SCI could provide active assisted stepping at a lower level of assistance that was provided by the exoskeleton.

Another attempt demonstrated the efficacy of TSS in combination with exoskeleton walking in a person with paraplegia [74]. The authors tested the hypothesis that TSS may complement the work of the exoskeleton by providing a motor drive and improve inter-limb coordination. The findings suggested that locomotor spinal networks can be neuromodulated with applications with non-invasive TSS [74]. Figure 2 presents unpublished pilot work that demonstrated that a person with complete motor SCI may neuromodulate the lumbosacral neural circuitry to generate active assisted steps when combining TSS with exoskeleton training. In this pilot work, the exoskeleton assistance was reduced by 5–10% and the subject was asked to actively generate steps during a 10 m walking test. The number of assisted (i.e., via the exoskeleton) vs. unassisted steps (i.e., actively assisted by the subject) were counted (Figure 2). The results indicated that the unassisted steps increased only when the TSS was turned on compared with no stimulation delivered (Figure 2).

The restoration of motor, sensory, and autonomic nervous system function after SCI has been the focus of many research groups in the last decade. This has led to several groundbreaking works that clearly demonstrated that the neuromodulation of the dormant spinal circuitry below the injury level may lead to the robust amplification of sensory/motor signals. The work was based on years of animal research that demonstrated that task-specific training or neuromodulation (epidural stimulation) may restore overground mobility even in the fully transected spinal cord [74]. The findings highlighted that spinal cord circuitry has the capability to integrate afferent sensory information and execute motor outputs independent of any supraspinal control [74,75]. A recent review has provided a detailed explanation on several potential mechanisms of the effects of epidural stimulation on the sensorimotor system in persons with SCI [75]. The works have gained considerable attention, especially considering the lumbosacral central pattern generators (CPGs). The CPG is a group of neural interneurons characterized by controlled rhythmicity and likely to contribute to several functions, including fictive locomotion, reflexive actions, reciprocal patterns between agonist and antagonists’ muscles, and, finally, spinal locomotion. This has led many researchers to recognize that the spinal cord is a perceptive central nervous system capable of learning, memorizing, and executing motor commands with task-specific training independent of any supraspinal control [75].

The use of epidural stimulation is being investigated for its ability to promote function in individuals with motor complete SCI and incomplete SCI [76,77,78,79]. Epidural stimulation involves the application of a continuous electrical current to the lower part of the spinal cord via an electrode implanted over the dura in the epidural space. When combined with task-specific movement practice, it can improve motor function [75,79,80]. A recently completed scoping review identified the potential benefits of epidural stimulation for individuals with SCI, including reductions in pain, spasms, and spasticity, improvements in the bowel, cardiorespiratory and cardiometabolic function, and an increased upper and lower extremity function [81]. Stimulation parameters varied for each study and for each study participant; however, for lower extremity function, stimulation sites ranged from T12 to S2 along the posterior cord. Jervis Rademeyer and associates promoted lower extremity function as an area with great promise in benefitting from epidural stimulation, especially with stimulation at the L2–L3 level. Positive benefits demonstrated included an increased time of independent standing and improved walking kinematics resulting in an improved walking speed [81].

Enrico et al. showed that task-specific training in participants with complete SCI could regain independent standing; however, starting locomotion training resulted in interfering with their standing capabilities [82]. It is interesting to note that even in participants with complete SCI, full overground ambulation has been restored with epidural stimulation. The mechanisms of how epidural stimulation manages to restore sensory–motor integration to restore locomotion have yet to be deciphered [75]. One of the explanations is sensory–motor integration via the continuous activation of the CPGs. Another explanation is the classification of complete SCI into dis-complete SCI, where limited axonal fibers from the pyramidal tract or other extra-pyramidal tracts successfully cross the injury site and reach the motor pools responsible for standing, stepping, and locomotion [75]. Applications of epidural stimulation are likely to amplify the sensory and motor signals and lead to motor behaviors with task-specific training.

There is emerging evidence that epidural stimulation may positively impact upper extremity function. The authors of [79] investigated the effects of a C5 stimulation site with a pulse width of 210 µs, frequency 2–40 Hz, and stimulation intensity between 0.1 and 10.0 mA in two individuals with cervical injuries. The results included an increase in grip strength during stimulation. Interestingly, some residual benefit in grip strength with the stimulation off was gained after a significant training period.

## 9. Neuromodulation of the Spinal Cord

The lumbosacral spinal cord contains a neural circuitry that can process proprioceptive and cutaneous information to generate cyclic motor patterns such as stepping [68]. The important aspect is that CPGs can spontaneously fire in the absence of supraspinal input, and they have a high level of automaticity [69]. The activity of CPGs is under the full control of the cerebral cortex, subcortical centers, and the brain stem. The rhythmicity of the CPGs is likely to induce a delicate balance between the extensors/flexors muscle groups that vary in a phase-dependent manner during the gait cycle [83]. Gerasimenko et al. successfully produced the stepping pattern while individuals were in a gravity-eliminated position by using non-invasive transcutaneous electrical stimulation to the spinal cord [69]. Surface electrodes were placed over the vertebrae of the lower thoracic and lumbosacral spinal cord. The electrical stimulation synapsed with interneurons that then stimulated neurons in the CPG circuitry.

The repeated activation of the sensory and motor systems through activity-based therapies has become an accepted strategy of enhancing neuroplasticity and restoring overground ambulation [75,81]. The mechanisms targeted to improve functional recovery after SCI are: (1) the modulation of axonal sprouting and synapse formation in spared and reorganized neural tissue, promoting axonal growth, (2) regeneration into and beyond non-neural lesion cores via placement of neural-stem-cell-derived grafts, (3) providing new relay neurons that can support host axon growth, and (4) the modulation of the sensory input steering circuit reorganization [81,84]. Individuals with complete SCI are limited due to the lack of intact excitable neural pathways. However, when excitable neural pathways remain intact after injury, plasticity-enhancing physical activities fostered by electrical stimulation are possible [75,81,84].

To highlight the possibilities of neuroplasticity after SCI, we reported on two individuals who participated in a series of electrical stimulation training protocols reported by [84]. The first participant was a 26-year-old male initially diagnosed with T6 motor and sensory complete SCI, and the second a 37-year-old male with T3 motor and sensory complete SCI. Injuries were sustained three and six years, respectively, before implantation with epidural stimulation. To confirm that physical activity alone would not restore motor function, both participants underwent 6 months of physical rehabilitation, including 60 motor training sessions over 22 weeks with rehabilitation specialists [77,78]. Each training session consisted of 15 min of stretching to the lower extremities, 45 min of locomotor training on a bodyweight-supported treadmill, and 30 min of balance and task-specific training. Afterward, a 16-contact epidural electrode array was inserted in the dorsal midline of the dura from T11 to L1. The participants were tested with FES attempting multiple motor tasks, including attempted standing, flexion, and extension leg movements while supine or side-lying. A variety of stimulation parameters were tested to optimize the stimulation during this time. Three weeks after the epidural implantation, the participants were tested in a side-lying position with the top leg suspended. Both participants were able to initiate, maintain, and terminate the electrically enabled leg activities [77,78].

Gill et al. determined the effects of interleaved epidural stimulation on the progression of stepping and overground locomotion in a person with complete SCI [85]. After implantation with a single paddle that was set at 210 µs, 25 Hz, and 5–6 volts, the participants underwent locomotor training with a 30% body weight support and a speed that was set 0.35 m/s. In week 4 session 11, till week 16 session 37, the participant still required maximum assistance at the hips and during the swing phase of the gait cycle. After switching to interleaved epidural stimulation in week 43, the combined frequency was set at 40 Hz (i.e., each paddle was set 210 µs, 20 Hz, and 3.3–3.7 volts), the participant was able to perform stepping at the treadmill with no required body weight support at a speed of 0.22 m/s, no assistance at the hips or the knees with arms supported on the treadmill bars for balance. Furthermore, the participant was to restore overground stepping with a front-wheel walker and with minimal assistance at the hips [85]. The authors also investigated the use of stimulation in the caudal region of the epidural array to improve the forward reaching ability of the participants. The participants were tested with and without electrical stimulation. Both participants were able to reach forward farther with either arm when the epidural stimulation was utilized [85,86].

Finally, Wagner et al. showed that targeted epidural stimulation can restore walking in persons with SCI [79]. The authors provided the multidirectional assistance of trunk movements to provide bodyweight support during overground locomotion with targeted epidural stimulation. Targeted mapping involved the activation of the upper lumbar segments and ankle extension mapping-involved activation of the upper sacral segments. Electrophysiological mapping was used to determine optimal electrodes and amplitudes for targeting specific proprioceptive circuits through the posterior. This resulted in shifting the paradigm from continuous epidural stimulation to more a temporal paradigm that was phase-dependent and coincided with different parts of the gait cycles. With spatiotemporal-targeted epidural stimulation, all participants with complete SCI restored voluntary control of overground walking [79].

## 10. Combined Epidural Stimulation and Exoskeletal-Assisted Walking

Gorgey et al. demonstrated the feasibility of using exoskeletal-assisted walking with epidural stimulation in an adult with a motor and sensory complete C7 injury. The exoskeleton provides robotic technology for overground weight-bearing ambulation with different levels of stepping assistance. This allows a low-metabolic cost for persons with SCI when walking with a 100% swing phase assistance for the legs [33]. With training, the amount of assisted stepping can be reduced depending on the degree of improvements developed by the participant. Gorgey and colleagues attempted to enhance this development by combining exoskeletal-assisted walking with an epidural electrical stimulation.

After epidural stimulator implantation along the spinal segments T12–S2, the participant underwent 12 weeks of exoskeletal-assisted walking with the first two weeks having 100% support with stepping [33]. The third week, the participant was transitioned to Canadian crutches from a walker. During weeks four through eight, swing phase assistance was gradually decreased depending on the participant’s performance. By week ten, the stepping or swing phase assistance had been decreased to 35% while the participant walked for at least 45 min per session. As the assistance level was decreased from 45% to 35% during the last weeks of the training, the number of unassisted steps also increased. Overall, through the 12 weeks of training (24 sessions), the participant decreased exoskeletal assistance from 100% to 35% and improved in temporal and rhythmic patterns as determined by electromyogram testing and gait speed [33].

**Figure 3:**
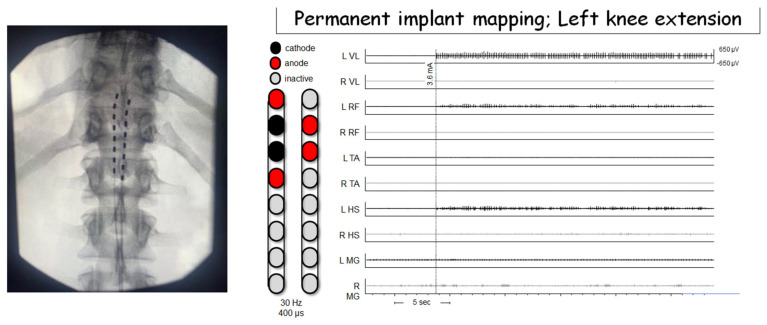
Epidural stimulation configuration for one participant resulting in left knee extension. All EMGs are shown on the same scale. Motor activity occurred at a stimulation amplitude of 3.6 mA. Phasic output is most prominent in knee extension muscles (L VL, L RF) with negligible activity at knee flexor muscles. Hz—hertz (stimulation frequency); µs—microseconds; L—left; R—right; VL—vastus lateralis; RF—rectus femoris; TA—tibialis anterior; HS—hamstrings; MG—medial gastrocnemius; sec—seconds; µv—microvolts.

Dissimilar to the paddle applications that may require a laminectomy, we are currently adopting the implantation of percutaneous leads (right and left) to activate the lumbosacral neural circuitries below the level of lesion. Each lead was mounted by eight contact electrodes that covered that distance from the T11 vertebra to the proximal border L1 vertebra. Figure 3 demonstrated successful mapping in a person with C8 cervical complete SCI in an attempt to target the muscle responsible for the initiation of standing and stepping.

## 11. Applications of Epidural Stimulation on Cardiovascular Performance

Epidural stimulation has been used for cardiovascular management in many populations for decades. The first studies showed that epidural stimulation, when delivered to the upper or mid-thoracic spinal cord, can alleviate symptoms of peripheral vascular disease [87,88] and improve baroreflex function in people with orthostatic issues [89]. Epidural stimulation was also shown to alter the response to an autonomic stressor, such as a cold pressor test [90].

**Figure 4:**
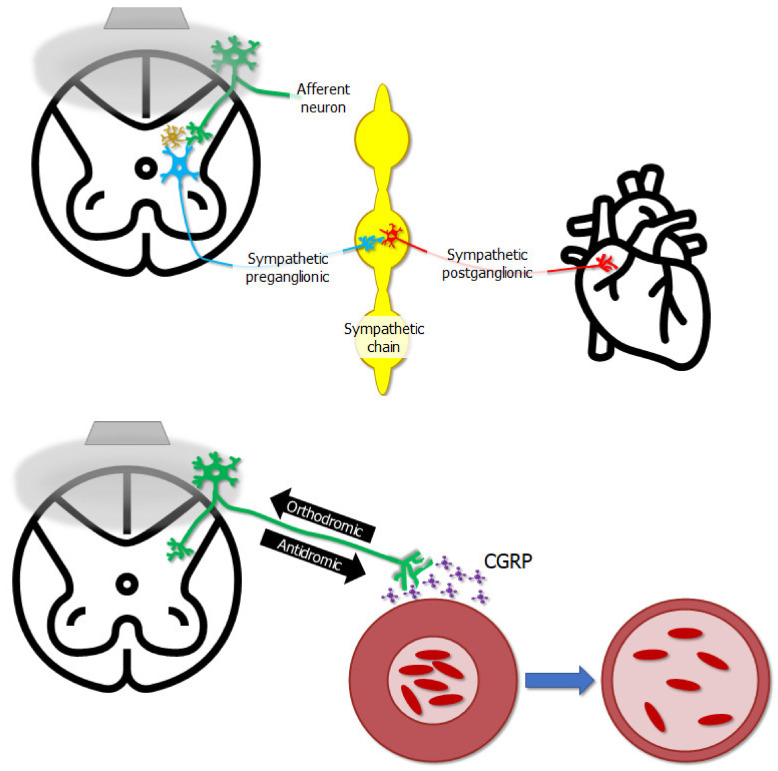
The gray trapezoid in the upper left represents a stimulator laying on the dorsal aspect of the spinal cord, and the gray bubble represents the electrical field emitted by the stimulator. The stimulator does not affect neurons innervating the heart directly—epidural stimulation affects the excitability of afferent neurons whose cell bodies lie in dorsal root ganglia on the dorsal aspect of the cord. These neurons in turn can affect the excitability of interneurons, represented by the gold neuron in the spinal cord, or afferent neurons can directly influence sympathetic preganglionic neurons. These in turn affect sympathetic post-ganglionic neurons, which finally innervate the heart, completing the chain of events by which epidural stimulation influences cardiac function. Electrical stimulation can potentially cause action potentials to propagate in an “antidromic” direction, i.e., the opposite direction of what is natural. In this case, action potentials in afferent neurons propagate from the periphery towards the spinal cord, as shown by the “orthodromic” arrow—however, epidural stimulation can push action potentials in the opposite “antidromic” direction, back towards the periphery. One outcome of this is the release of calcitonin gene-related peptide (CGRP) near blood vessels, causing vasodilation and altering local blood flow.

Studies using animal models have provided mechanistic insight into how epidural stimulation can relieve angina pectoris [91,92], cause vasodilation [93], or also relieve orthostatic hypotension [94], supporting its use clinically. One of the ways cardiac function may be affected is that epidural stimulation seems to exhibit an indirect effect on the heart’s intrinsic nervous system [91] (Figure 4). Another way cardiovascular function is affected by epidural stimulation is via the antidromic depolarization of afferent neurons leading to the release of calcitonin gene-related peptide, causing vasodilation [93] (Figure 4). While there is some fundamental mechanistic insight into how epidural stimulation can affect cardiovascular function, there are also unique considerations for how epidural stimulation affects cardiovascular function in persons with SCI, and the mechanisms explaining subsequent effects.

Epidural stimulation has evolved in the last decade to be a promising approach to facilitate or restore motor functions lost after SCI, even in individuals with severe, motor complete injuries [75]. A critical point is that these results have been shown with epidural stimulation to the lumbar spinal cord, not to the thoracic cord as with previous cardiovascular literature. Despite this, lumbar epidural stimulation has shown many promising results for cardiovascular function in persons with SCI. For instance, even in persons with clinically complete SCI, epidural stimulation can induce favorable changes in blood pressure regulation and the baroreflex response to postural changes [95,96,97], improve heart rate variability [98] enable improved cognitive function during an orthostatic challenge [99], improve cardiac output [95], and across a period of combining physical training with epidural stimulation, can favorably remodel cardiac tissue [100]. Theoretical mechanisms explaining these changes have been proposed in the literature [95,101].

**Figure 5:**
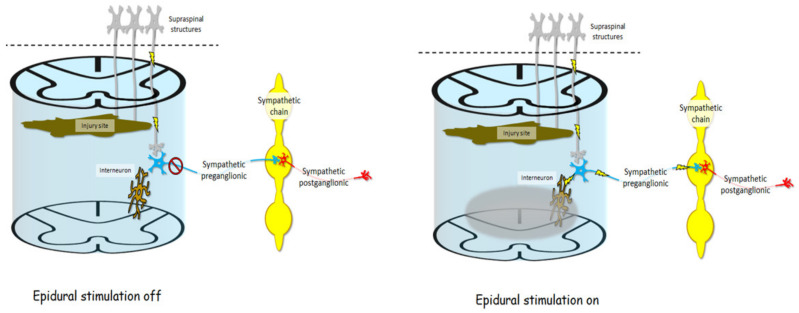
After SCI, axons descending from supraspinal neurons, such as those in the rostral ventrolateral medulla, may be cut off completely by the injury site, or spared connections may exert so little influence at the spinal level that sympathetic preganglionic neurons are dormant and therefore do not exert any influence on the sympathetic chain, and therefore do not influence sympathetic postganglionic neurons or organs subserving cardiovascular functions. Electrical fields delivered by epidural stimulation (gray bubble) may indirectly influence multi-segmental interneurons via dorsal root afferents (not pictured). This influence on interneurons may change the baseline excitability of sympathetic preganglionic neurons, making them more sensitive to any input from afferent or descending neurons, thereby enabling them to fire and in turn excite postganglionic neurons in the sympathetic chain, resulting in better cardiovascular regulation.

Figure 5 shows a visual model of an injured spinal cord and sympathetic structures influencing cardiovascular function without and with epidural stimulation, respectively. After SCI, brainstem neurons which help regulate cardiovascular control may be cut off from their target neural circuits in the spinal cord. While many clinically complete injuries are not anatomically complete [102], even if descending signals can get past the injury, they may be too weak to exert any influence on surviving sympathetic preganglionic neurons. If those neurons are inactive, they will not influence the sympathetic chain, and sympathetic postganglionic neuron exertion to cardiovascular organs will be reduced, thereby giving rise to cardiovascular dysregulation. However, when epidural stimulation is delivered to the lumbar spinal cord below the injury it can act through dorsal root afferents to influence interneurons in the spinal cord which can span multiple spinal segments, subsequently exerting influence on sympathetic preganglionic neurons [95,101]. Once brought into a more excitable state, sympathetic preganglionic neurons are then enabled to influence the postganglionic neurons originating in the sympathetic chain, thereby influencing cardiovascular function in ways that have been beneficial to persons with SCI.

## 12. Summary and Conclusions

This review article presented the latest evidence on targeting function with invasive and non-invasive approaches using electrical stimulation in people with SCI. Clinicians should consider this evidence-based information, including the clinical applications of FES/NMES to optimize its use for different treatment goals (Table 1). The routine uses of FES/NMES in combination with other therapies, such as standing frames and treadmill training, could maximize muscle health, aerobic fitness, and cardiometabolic outcomes.

Some of the limitations that prevent the routine use of FES/NMES in clinical practice have been reported as a lack of confidence and acceptance from clinicians and patients and the expenditure of time and money [103]. Thus, the dissemination and/or implementation of training modules for clinicians, patients, and clinical and governmental organizations that determine the funds allocated for the implementation of these therapies should be considered. Other considerations to facilitate the use of NMES/FES should be the use of portable and inexpensive devices that could be used as home therapy. These can be implemented with add-on training packages that would target the lack of confidence and acceptance of these devices from clinicians and patients. This would allow the closure of clinical practice and policy development gaps identified in the literature [49].

Another important identified gap is the lack of research quality on the effects of FES cycling in the following outcomes: pressure injuries, chronic pain, urinary tract infections, and subjective well-being [49]. These may be addressed effectively if research designs are robust in quality and if a patient-centered approach is being implemented systematically in all research designs targeting benefits in people with SCI [104,105]. Developing integrated knowledge translation guiding principles when conducting and disseminating research in people with SCI, involving the research users from inception till dissemination of research results, seems to be the appropriate approach for future research development in people with SCI [105].

While larger clinical trials are warranted, emerging evidence demonstrates great potential in using invasive, less invasive, and non-invasive electrical stimulation activities for enhancing the functional abilities of those with SCI. Whether it be through NMES, FES cycling, or task-specific movement training enhanced by epidural electrical stimulation, the benefits of using invasive and non-invasive electrical stimulation for targeting function have the potential to enhance functional mobility and increase the quality of life of people with SCI.

## Figures and Tables

**Table 1:** Summary of the major findings that reflect key studies in non-invasive and invasive applications of electrical stimulation to enhance functions after SCI.

Approaches of Delivering Electrical Stimulation	Studies	Major Findings
Non-invasive Approaches	Gorgey et al. [5]	NMES plus testosterone group significantly increased in total body lean mass, whole muscle mass, and knee extensor cross-sectional area and accompanied with increase in basal metabolic rate.
	Demchak et al. [35]	The cross-sectional area of the vastus lateralis muscle increased 63% more than the non-stimulated leg.
	Dolbow et al. [36]	A 5.7% increase in leg lean mass and a 2.4% decrease in BF% following 8 weeks of high-intensity FES training.
	Fornusek et al. [37]	Increase in thig girth following six weeks of low-cadence FES cycling
	Gorgey et al. [40]	Spacing the frequency of the sessions per week or limit the sessions to 2x per week may be effective strategy to reduce low frequency fatigue.
	Bochkezanian et al. [42]	Higher intensity resistance training produces strong muscle contractions and significant gains in muscle strength and muscle mass with improvement in reported measures of spasticity after SCI.
	van der Scheer et al. [49]	FES cycling has a moderate–high certainty for improvements in muscle health, referring to muscle mass, fiber-type composition.
	Houston et al. [57]	A closed-loop FES system was recently combined with visual feedback to improve standing balance.
	Vette et al. [63]	FES can facilitate completion of visual feedback balance training by targeting the dorsiflexor and plantar flexor muscles.
	Gauthier et al. [64]	The feasibility of incorporating FES into perturbation-based balance training to ensure retraining-reactive postural control.
Less Invasive approaches		
	Gerasimenko et al. [69]	Stepping pattern in a gravity-eliminated position by using non-invasive transcutaneous electrical stimulation to the spinal cord.
	Benavides et al. [70]	TSS had an excitatory effect at the spinal level as measured by cervico-medullary-evoked potentials and an inhibitory effect at the cortical level as measured by motor-evoked potentials.
	Gad et al. [73]	After eight training sessions with TSS, combined with training over four weeks for 1–2 h per session, maximum voluntary handgrip forces increased by 325% in the presence of stimulation and 225% when grip strength was tested without stimulation in chronic tetraplegia.
	Gad et al. [74]	TSS may complement the work of the exoskeleton by providing motor drive and improve inter-limb coordination. The findings suggested that locomotor spinal networks can be neuromodulated with applications with non-invasive TSS.
Invasive approaches		
	Enrico et al. [82]	Task-specific training could regain independent standing; however, starting locomotion training resulted in interfering with their standing capabilities.
	Harkema S et al. [77] Angeli et al. [78]	Participants were able to initiate, maintain, and terminate the electrically enabled leg activities after implantation.
	Wagner et al. [79]	Targeted epidural stimulation can restore walking in persons with SCI.
	Gorgey et al. [33]	Exoskeleton stepping or swing phase assistance decreased to 35% while the participant walked for at least 45 min per session with epidural stimulation.
	Galley et al. [87]	Epidural stimulation, when delivered to the upper or mid-thoracic spinal cord, can alleviate symptoms of peripheral vascular disease.

## Data Availability

Not acceptable.
